# The landscape of microsatellites in the enset (*Ensete ventricosum*) genome and web-based marker resource development

**DOI:** 10.1038/s41598-020-71984-x

**Published:** 2020-09-17

**Authors:** Manosh Kumar Biswas, Jaypal N. Darbar, James S. Borrell, Mita Bagchi, Dhiman Biswas, Gizachew Woldesenbet Nuraga, Sebsebe Demissew, Paul Wilkin, Trude Schwarzacher, J. S. Heslop-Harrison

**Affiliations:** 1 Department of Genetics and Genome Biology, University of Leicester, Leicester, LE1 7RH UK; 2 Royal Botanic Gardens, Kew, Richmond, TW9 3AE Surrey UK; 3 Department of Computer Science and Engineering, Maulana Abul Kalam Azad University of Technology, Kolkata, West Bengal India; 4 Institute of Biotechnology, Addis Ababa University, Addis Ababa, Ethiopia; 5 Department of Plant Biology and Biodiversity Management, Addis Ababa University, Addis Ababa, Ethiopia; 6 South China Botanical Garden, Chinese Academy of Sciences, Guangzhou, People’s Republic of China

**Keywords:** Genetic markers, Genotype, Plant breeding, Plant genetics

## Abstract

*Ensete ventricosum* (Musaceae, enset) is an Ethiopian food security crop. To realize the potential of enset for rural livelihoods, further knowledge of enset diversity, genetics and genomics is required to support breeding programs and conservation. This study was conducted to explore the enset genome to develop molecular markers, genomics resources, and characterize enset landraces while giving insight into the organization of the genome. We identified 233 microsatellites (simple sequence repeats, SSRs) per Mbp in the enset genome, representing 0.28% of the genome. Mono- and di-nucleotide repeats motifs were found in a higher proportion than other classes of SSR-motifs. In total, 154,586 non-redundant enset microsatellite markers (EMM) were identified and 40 selected for primer development. Marker validation by PCR and low-cost agarose gel electrophoresis revealed that 92.5% were polymorphic, showing a high PIC (Polymorphism Information Content; 0.87) and expected heterozygosity (He = 0.79–0.82). In silico analysis of genomes of closely related species showed 46.86% of the markers were transferable among enset species and 1.90% were transferable to *Musa*. The SSRs are robust (with basic PCR methods and agarose gel electrophoresis), informative, and applicable in measuring enset diversity, genotyping, selection and potentially breeding. Enset SSRs are available in a web-based database at https://enset-project.org/EnMom@base.html (or https://enset.aau.edu.et/index.html, downloadable from Figshare).

## Introduction

*Ensete ventricosum* (Musaceae) is a giant monocarpic perennial herbaceous plant, wide spread in tropical East and Southern Africa, and domesticated in Ethiopia, where it provides the main starch staple for 20 million rural people^1^. It is known as Ethiopian, Abyssinian or false banana, ensete, or (as used here) enset. The genus extends from Africa to tropical eastern Asia, and is wild as well as cultivated in Ethiopia, a Vavilovian centre of plant diversity^2,3^. While banana and plantain (*Musa* spp.) are cultivated for their fruits, enset starch is extracted from the pseudostem, leaf sheaths and underground corm. Enset is harvested year-round, and is reportedly drought tolerant^4^ so is known as a food-security crop^5^. With good management and relatively low inputs, production per unit area is higher than most cereals^6^, so it can feed a large population^7^. Enset plants store starch, reaching a maximum shortly before flowering, and 40 kg starch can be harvested from 3 to 4 year old plants. Enset is also good sources for fibre, medicines and animal fodder and the leaves may be used for packaging, fibre, and roofing^8^.

Several thousand landraces with high genetic diversity have been reported in banana^9^ worldwide. For enset, multiple morphologically distinct landraces are grown in each small holding, and DNA markers^10^ show they are diverse. The diversity of the few hundred enset landraces, with local names, is now being surveyed from different agro-ecological regions in Ethiopia^10,11^. There are several enset germplasm collections maintained in Ethiopia, including Hawassa University, Wolkite University, Southern Agricultural Research Institute (SARI)^12,13^ and Ethiopian Biodiversity Institute (EBI)^14^.Unlike triploid banana, the diploid enset is able to produce plants from the seeds of its non-edible fruits, although most enset grown on farms is not permitted to flower and is propagated clonally. Enset genetic resource conservation and management rely on cultivation with regular renewal. Vegetative propagation, vernacular naming systems, and the long juvenility period makes enset improvement or breeding difficult and expensive^15^.

With the knowledge gap about enset genetics, distribution and diversity, several studies have been conducted to estimate genetic diversity and define relationships among the limited enset germplasm stocks. Most of these studies use DNA markers including Random Amplified Polymorphic DNA (RAPD)^16^, Inter-Simple Sequence Repeats (ISSR)^11^, and Amplified Fragment Length Polymorphism (AFLP)^17^, although some of these marker techniques are high-cost, show limited reproducibility, or identify only dominant alleles (from a heterozygous crop without systematic inbreeding). Simple sequence repeats (SSR) were identified by using a CT and GT repeat-enriched pyrosequencing (454) library by Temesgen et al.^10^, who tested 217 pairs of microsatellite primers, of which 67 showed amplification; 59 were polymorphic and 34 were published and used for their analysis^10^.

There is minimal breeding of enset as a crop, although genetic diversity analysis shows that cultivated accessions are genetically different to most wild accessions^5,18–20^; there is the opportunity for future application of marker-assisted breeding^10^ and marker based parental choice in crossing programmes in the crop. Genetic diversity and population structure studies are required for enset in Ethiopia for germplasm management, identifying landraces or cultivars, collection-management, and determining phylogenetic relationships. Genotyping-by-sequencing and transcriptome sequencing (RAD-seq or RNA-seq), and including SNP (Single Nucleotide Polymorphism) analysis, provide the deepest and highest coverage of genomic diversity, but both experimental and analytical costs are very substantial.

Microsatellites (SSRs, along with single-locus PCR markers such as cleaved amplified polymorphic sequence) are robust, have adequate genome-wide coverage for most targeted purposes, are relatively low cost, and can be used informatively on a small or large number of accessions. Development of large numbers of microsatellite markers—thousands to tens of thousands—from genomic DNA sequences is possible. Their applications include genetic diversity surveys^21–25^, population structure analysis^26,27^, genotyping^28–30^, association mapping^31–35^, linkage mapping^36,37^ and ultimately plant breeding. Microsatellite markers are usually robust with less dependence on DNA quality and laboratory environment, require only basic molecular-biology equipment (here, aiming to find polymorphisms detectable by agarose gel electrophoresis), are co-dominant, and often are transferable to related species. Draft whole genome sequences of *Ensete ventricosum* are available in the public domain^38,39^, although so far newly isolated sequences or transferable banana microsatellite markers have been used for enset genetic diversity study^40^. Large microsatellite databases have been developed for many crop plants^41,42^.

The present study aimed to exploit the draft whole-genome sequence of enset to (1) Identify microsatellite sequences and characterize their genome-wide landscape, including the nature of motifs, frequency, genomic distribution and, where appropriate, functional annotation; (2) Identify candidate primers for all microsatellites in a large-scale microsatellite database, and develop web-based open tools for access; (3) Validate a subset of candidate microsatellite primers both in silico and by PCR amplification of isolated DNA and fragment analysis; (4) Compare the genome-wide microsatellite landscape in enset with *Musa* species, assessing cross-taxa transferability both in silico and by PCR; (5) Recommend a sub-set of markers use for genetic diversity analysis.

## Results

### Microsatellite content in enset and cross-species comparisons

**Figure 1:**
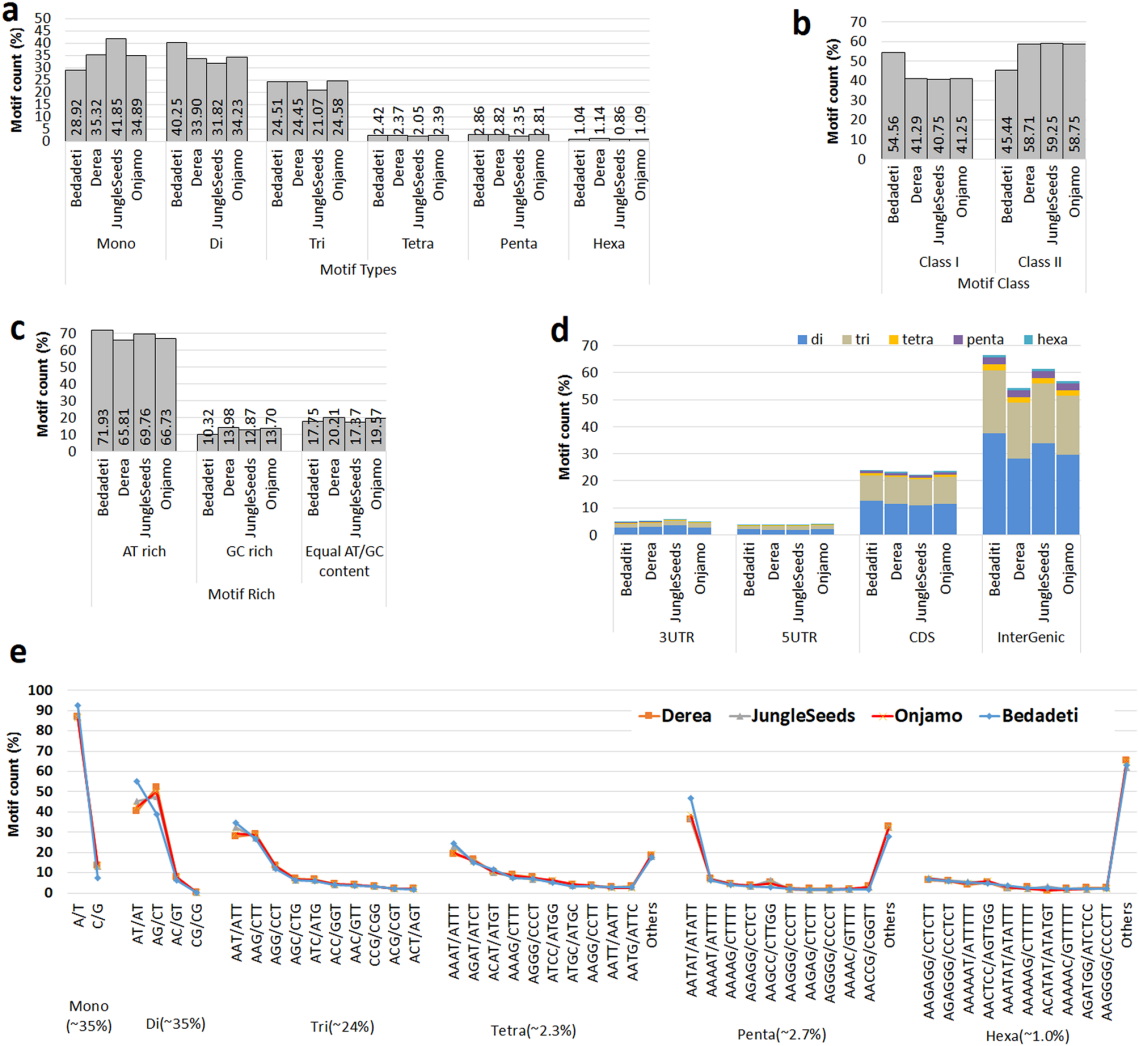
Comparative microsatellite frequency distribution in four *Ensete ventricosum* genomes. (**a**) Motif type distribution, (**b**) motif class (Class I > 20 bp and Class II ≤ 20 bp) distributions, (**c**) motif distribution by nucleotide base composition (balance motif rich = motif compose equal ration of AT and GC content), (**d**) motif distribution among different genomic regions, (**e**) distribution of mono-hexa nucleotide repeat motifs.

The pipeline for identification of microsatellites in whole genome sequences of four published enset landraces (*Ensete ventricosum*, ‘Bedadeti’ ‘Derea’ ‘Onjamo’ and ‘Jungle Seeds’), is shown in Fig. S1 and the complete data are given in the enset database at https://enset-project.org/EnMom@base.html. The database can be downloaded from https://figshare.com/s/20dd8c0d0a2994dbce8d with CC-BY-4.0 licence). Between 93,000 and 115,000 microsatellites were detected in the genome assemblies (Fig. 1, Tables 1 and S1), with an average microsatellite density of 233 per Mb (Table S1). Mono-, di-and tri- nucleotide repeats were frequent, with fewer tetra-, penta- and hexa- nucleotide repeats (Fig. 1a). Microsatellites were classified^43^ with the longer class I (> 20 nt) slightly more frequent that class II (≤ 20 nt) (Fig. 1b). AT-rich microsatellites were seven-fold more frequent than GC-rich microsatellites or those with equal AT/GC content (Fig. 1c).

**Table 1:** Primer modelling and in silico characterization summary of the enset microsatellite markers.

	Bedadeti	Derea	JungleSeeds	Onjamo	Total
Microsatellites identified	105,347	93,180	115,315	97,338	411,180
Primer modelling successful	68,559	52,570	51,844	55,699	228,672
Primer modelling success rate (%)	65.08	56.42	44.96	57.22	55.61
Non redundant primers	619,58	49,258	47,399	51,789	210,404 (154,586)*
Redundant primers (%)	10.12	6.39	9.03	7.04	27.41
Number of polymorphic markers^a^	41,531	36,923	33,047	38,284	37,446
Number of transferable markers^a^	52,112	46,305	36,979	48,369	45,941

*Number of single unique primer pairs, obtained after clustering 210,404 primer pairs.

^a^Results obtained from the e-PCR analysis.

Flanking regions (about 200 bp each side) of microsatellites were extracted and classified into intergenic regions (non-coding), coding regions and 3′ or 5′ UTRs. Microsatellites were over-represented near predicted coding sequences (24% of sequences compared to 16% in the entire genome) (Fig. 1d). The most common mono- to hexa-nucleotide microsatellite types in the *E. ventricosum* genome, are shown in Fig. 1e and Table S2.

Microsatellite content of *E. ventricosum* was compared with fourteen monocot and one gymnosperm species where sequence data are available using with similar microsatellite discovery methods (Tables S1 and Fig. S2). The microsatellite density (233 SSRs/Mbp and 0.28% of the genome) in *E. ventricosum* genome was similar to the four *Musa* species (average 207 SSRs/Mbp), and higher than other studied monocots (28–198 SSRs/Mbp, except *Spirode lapolyrhiza* (385 SSRs/Mbp; Table S1). Like the *Musa* species, most microsatellites in enset were AT-rich (67% vs. 69%; Table S1).

### Marker development, functional annotation, cross-taxa transferability and comparative mapping

An automated strategy successfully designed primers in about half of the SSR flanking sequences. Primer redundancy or non-specificity often arises from duplicated regions within genomes, and we used a Perl-based script to eliminate redundant primers (6.39–10.12%), leaving a total of 210,404 unique primer pairs (Table 1). As shown above, there were no notable differences in microsatellite frequency between the four landraces, and clustering showed that 27% of primers were identified in two or more landraces, giving 154,586 unique primers (Table 1) deposited in the Enset Microsatellite Marker (EMM) database (at https://enset-project.org/EnMom@base.html). We found that 20 of the new unique microsatellite markers coincided with the 217 identified by targeted sequencing of a CT- and GT-enriched library^10^ (Table S3).

In silico comparative mapping of the enset marker amplicons to genomes of four *Musa* species revealed that 19,579 (12.67%; range 12.44–12.92%) were found in each *Musa* species (Table S4). The high-quality, chromosome-level assemblies of three *Musa* genomes showed wide spread distribution of the enset markers with 307–573 (average 435 ± 74) allocated to each *Musa* chromosome, and some clustering around the putative centromere (Table S4 and Fig. S3).

**Figure 2:**
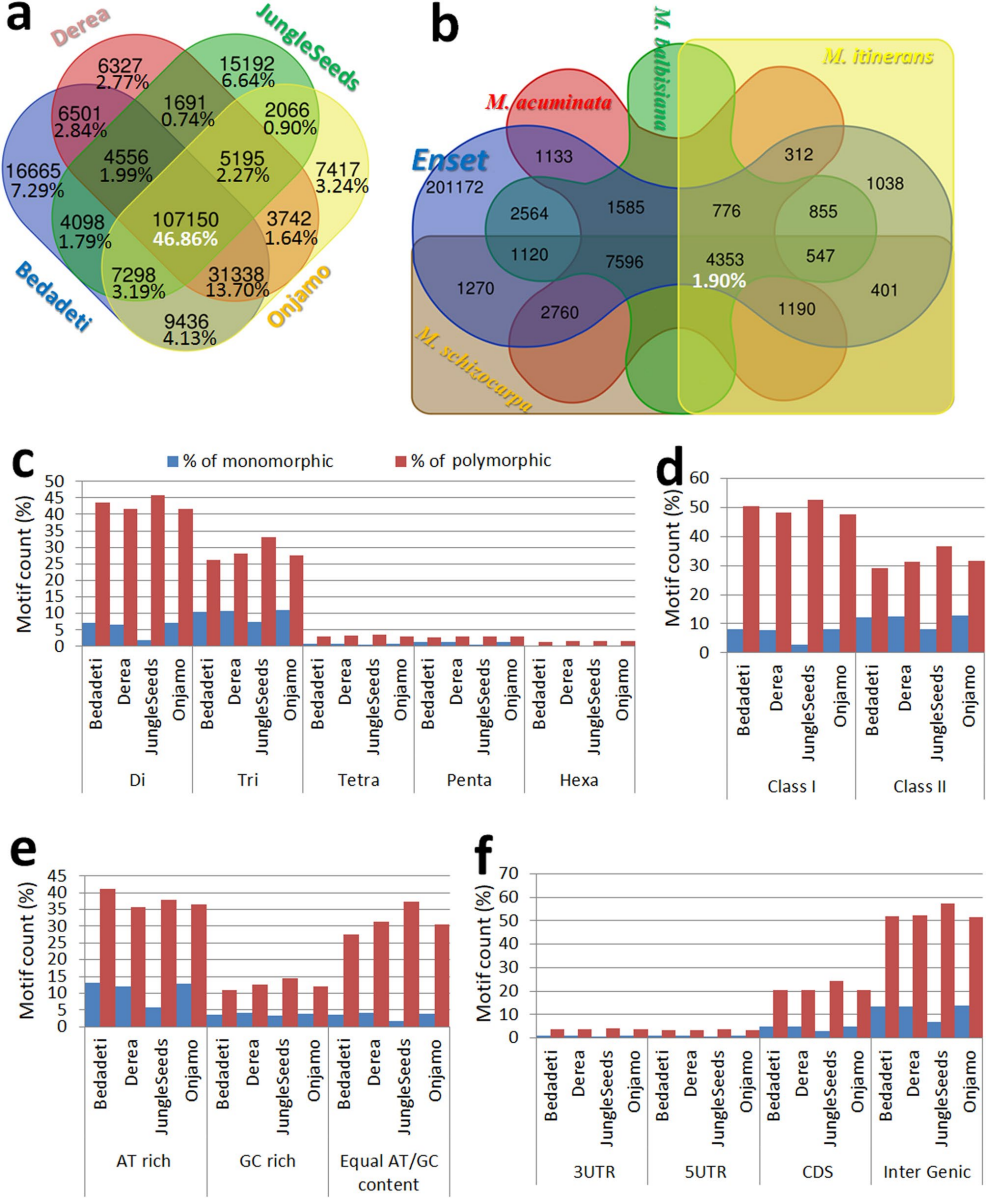
Venn diagram showing the number of common and specific EMM markers in (**a**) enset accessions and (**b**) *Musa* species. In silico characterization summary of the EMM markers (**c**–**f**).

In silico PCR^44^ was used to explore transferability and polymorphisms of the microsatellite markers across the four enset landraces and three *Musa* species (Table 1; Tables S5). Between 3 and 7% of markers were landrace-specific (‘Bedadeti’, ‘Derea’, ‘JungleSeeds’ and ‘Onjamo’) and about half were common across the four genomes (Fig. 2a). Only 4353 (1.90%) were transferable to the four *Musa* genomes (Fig. 2b), mostly di- and tri-nucleotide (> 20 bp, class I) repeats. Analysis of in silico PCR polymorphisms showed that 83% of the markers were polymorphic, with slightly higher polymorphism frequencies among longer (class I), di-nucleotide, and AT or GC-rich microsatellites (Fig. 2c–f).

### Marker validation and genetic diversity

**Figure 3:**
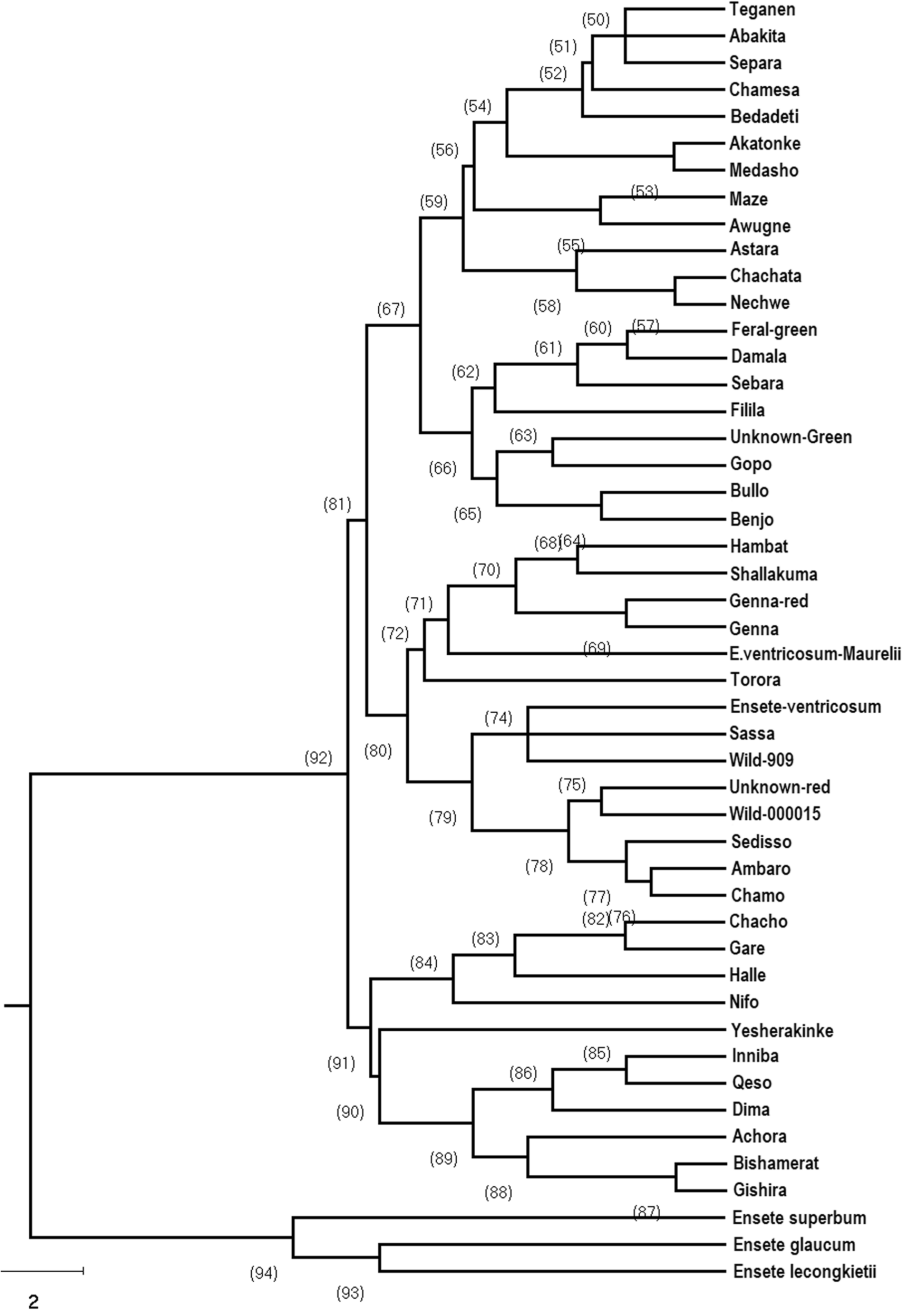
Phylogenetic relation analysis of 45 *Enseteventricosum* landraces and three *Ensete* species. “Ensete-ventricosum” and “E-venticosum Maurelii” are commercially available in the UK. “Unknown Red” was a feral plant.

The 154,586 unique markers were filtered in silico to select monomorphic primers, those with functional annotation, and transferability to enset relatives (Tables S5, S6). From a sample of 40 primer pairs used for PCR amplification using DNA from four enset and one *Musa* samples (Fig. S4), 34 (85.0%) gave the expected primer size, of which 33 were polymorphic (Table S7). In total, 126 polymorphic alleles were scored with a range of 1–6 per locus and an average PIC value of 0.87. The expected heterozygosity (H_e_) ranged from 0.79 to 0.82, while the observed heterozygosity (H_o_) ranged from 0.41 to 0.63 (Table S7). From these primers, 15 markers were chosen for genotyping 45 *E. ventricosum* wild, cultivated and landrace germplasm collections as well as three related species *E. superbum*, *E. glaucum* and *E. lecongkietii* (Fig. S5 and Table S8). The H_e_ for all accessions was 0.48. The mean F (Fixation Index) value indicated moderate to high genetic differentiation between species (0.36) (Table S9). The AMOVA analysis for distinguished *Ensete* species are presented in Table S10, and result reveals that AMOVA analysis enabled some clustering of enset landraces by their genetic variation. Neighbor-joining phylogenetic analysis strongly supported the 45 *E. ventricosum* accessions as a sister group to the three other *Ensete* species, well-resolved with bootstrap values > 91%. Within the *E. ventricosum* accessions, there was weaker bootstrap support for a few phylogenetic groupings of accessions (Fig. 3).

### Enset microsatellite database architecture, features and utility

**Figure 4:**
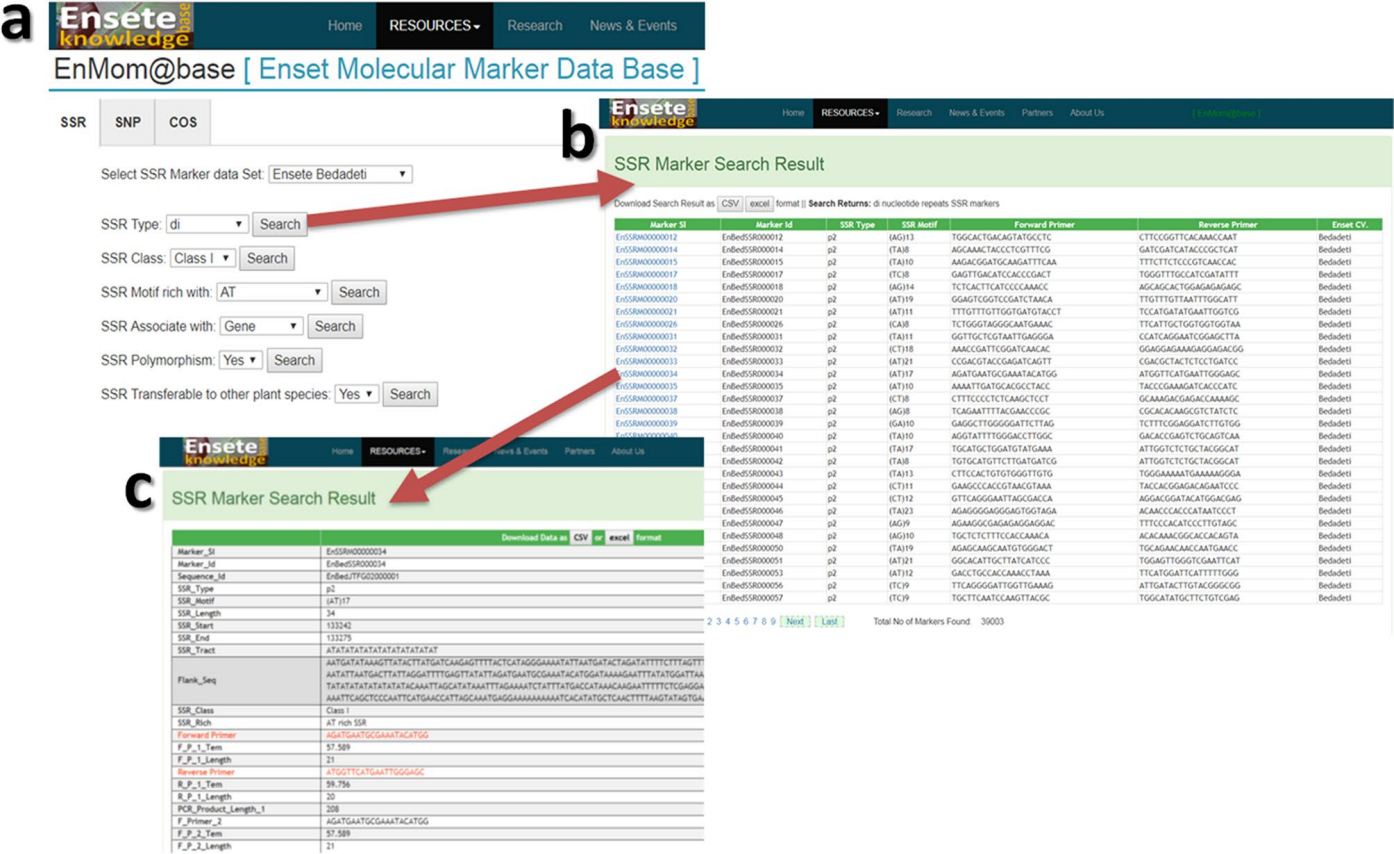
The online Enset Microsatellite Marker (EMM) database. (**a**) Enset microsatellite marker search page, (**b**) search result, (**c**) linked page for detail information of each EMM.

The enset microsatellite marker (EMM) information has been placed in the EMM-database (https://enset-project.org/EnMom@base.html) including search fields for microsatellite type, length, motif type, transferability, polymorphisms, and name (Fig. 4). The search returns a list of markers with Marker ID, microsatellite type and motif, forward- and reverse-primer sequences, and the name of source landrace, with links to additional information including genome position, transferability, predicted polymorphism, PCR product size, flanking sequences, any functional annotations, three sets of primer sequences and annealing temperatures. Query results can be downloaded in XLS and CSV file format for subsequent use.

## Discussion

Analysis of microsatellites in enset defined microsatellite numbers, motifs and polymorphisms, and allowed development and testing of a genomic resource of microsatellite markers for landrace identification and analysis of diversity in the species and its relatives. The microsatellite analysis pipeline found an average of 233 SSRs per Mb, within the range known for both monocotyledonous and dicotyledonous species^43–46^. As expected, we did not find significant differences in microsatellite occurrence between the four enset landraces across our analyses. As in a range of monocotyledons, dicotyledons and a gymnosperm, mono-, di- and tri-nucleotide microsatellite motifs in enset represented the majority of all motifs between 1- and 6-bases long. Despite reanalysis of the abundance of microsatellite motifs in genome assemblies of four *Musa*, eight grasses, two other monocots and a gymnosperm, there were few other notable patterns in the abundance of different motif lengths. Tri-nucleotide repeats are twice as frequent as 1- and 2-bp repeats in Poaceae species (except wheat and barley), while di-nucleotide repeats are twice as frequent as 1- and 3-bp repeats in Musaceae. Enset also has a low frequency of 3-bp repeats, with an equal frequency of 1- and 2-bp repeats.

Various approaches to microsatellite identification have been used, including sequencing microsatellite-targeted libraries (e.g.10 in enset), analysis of EST (expressed sequence tag) or RNA-sequence results, analysis of BAC or BAC-end-sequence (GSS, genomic survey sequences), and analysis of whole-genome assemblies. Whole genome sequences use various approaches (technology, depth, length, gap-filling) with widely different N50 values and proportions of reads left unassembled. There are technical limitations in assembly only based on short parid-end reads (Illumina), so repetitive regions (with satellite repeats or transposable elements) will not be assembled, and both microsatellites and primers will normally be included in single read pairs, so longer SSR stretches will be omitted or wrongly assembled. Thus assemblies around the microsatellites analysed here will lie mostly in low-copy-number genomic regions. Previous comparative analyses with diverse plant species including not only *E. guineensis*^47^, bamboo^48^, Triticeae cereal species^49^, and foxtail millet^50^, but also sweet orange^51^, *Nicotiana*^52^, cucumbers^53^ and others also have reported different relative abundances of mono-, di- and tri-nucleotide repeats. Victoria et al.^54^ reported that di-nucleotide motifs were more frequent in green algae and mosses, with tri-nucleotide repeats being more abundant in monocots and dicots; we note that di-nucleotide motifs also seem more abundant in enset and *Musa* species, as well as *Spirodela* and *Picea* (Table S1), so there is no clear correlation with evolutionary position nor phylogenetic branch, important in building a picture of SSR evolution across all plants.

Grouped motifs A/T, AT/AT, AAT/ATT, AAAT/ATTT and AATAT/ATATT were the major microsatellites in enset. AT-rich microsatellite were also abundant in other monocot species such as *Musa* spp., *Sorghum bicolor, Elaeis guineensis, Phyllostachys edulis,* and *Piceaabies* (Fig. S2). In contrast, some studies have found that AT-rich repeats were dominant in dicots and CG-rich repeats in monocot plant species^53,55^, so we suggest that base compositions of the most abundant microsatellites are not related to taxon nor genome sizes. A study of correlations of AT/GC-richness of microsatellite motif with genome-wide content^51^ demonstrated that AT/GC-richness was correlated with the genomic AT/GC-content, supported by our results. We report that more than 60% of microsatellite loci were located in the intergenic regions of enset genome supporting comparative data^56^.

For development of usable markers from the microsatellite survey, we found 56% of the loci were appropriate for primer modeling, similar to other genome-scale marker development studies^45,46,50,51,57^. We identified 27% of primer redundancy, some what higher than the 5–20% in other genome scale analyses in plants^25,51^, most likely reflecting an artifact of assembly where one primer-SSR read is mismatched with more than one SSR-primerread^58,59^. Cross-taxon transferability features eg Refs.^49,54^ facilitate comparison of multiple taxa, gene mapping, and identification of orthologous loci including discovery of gene polymorphisms. In silico analysis showed about half the *E. ventricosum* markers were transferable to three other *Ensete* species, but only 1.90% were transferable to all the four *Musa* genomes.

Marker validation by PCR showed that the in silico testing strategies were able to select markers with successful amplification and high levels of polymorphism. The 93% of those tested were polymorphic, similar to the percentage reported using a DNA microsatellite library in enset^10^ although our genome-wide selection had a higher average PIC value showing they are informative markers for population genetic studies. The mean expected heterozygosity (He = 0.80) was also higher than previously reported in enset microsatellites (He = 0.59)^10^ and eleven cross-transferred *Musa* SSRs (He = 0.55)^40^. The in silico selection strategy confirmed by the validation shows that the markers are robust and require little laboratory-based optimization for scoring polymorphisms by agarose gel electrophoresis, contrasting with RAPD, AFLP, ISSR^10,16,17^, which also showed lower levels of polymorphisms.

The AMOVA analysis distinguished *Ensete* species, and enabled some clustering of enset landraces by their genetic variation. This result is consistent with several studies^10,17,40^: for example AFLP marker based genetic diversity of 146 enset landraces from five geographical regions showed a small variation among geographical regions (4.8%), but a high variation (95.2%) within regions^17^. The PIC values in enset are similar to those reported in other species such as *Aegilops*^60^ although notably the enset heterozygosity was high (average Ho = 0.87), suggesting outbreeding among the parents, and contrasting with the low level seen in the largely inbreeding *Aegilops*. The phylogenetic relations of *Ensete* landraces use in this study in general agree with patterns in another group of landraces analysed in the SSR-based phylogeny of Olango et al.^10^. In this study we found that the clustering pattern of landraces of *E. ventricosum* does not reflect any division based on cultivation region. These findings further confirm the extensive practice of germplasm (young plant or sucker) exchange between enset growers communities.

The markers derived from the sequence analysis are freely available in the EMM-database (https://enset-project.org/EnMom@base.html) and the whole database can be downloaded from https://figshare.com/s/20dd8c0d0a2994dbce8d. Microsatellite marker databases have been developed for various crop plant species including foxtail millet^41^, eggplant^61^, tomato^42^, oil palm^62^. The Enset Marker database and contains flexible search and download features, enabling large numbers of markers to be extracted and providing a resource for enset crop improvement. Many hundreds of additional markers can be extracted for targeted genotyping, GWAS (genome wide association studies) or marker assisted selection (MAS) studies as well as genetic diversity analysis, understanding population structures, and landrace identification.

## Materials and methods

### Genome-wide microsatellite exploration, characterization and marker development

We explored the microsatellite landscape using the whole nuclear genome sequence assemblies of four enset landraces (*E. ventricosum* ‘Bedadeti’ GenBank assembly: GCA_000818735.2; *E. ventricosum* ‘JungleSeeds’ GenBank assembly: GCA_000331365.2; *E. ventricosum* ‘Onjamo’ GenBank assembly: GCA_001884845.1; *E. ventricosum* ‘Derea’ GenBank assembly: GCA_001884805.1). The microsatellite mining pipeline was built with a combination of open bioinformatics tools including MISA (https://pgrc.ipk-gatersleben.de/misa/), Primer3^63^, e-PCR^44^ (in silico PCR) and Perl-scripts (Fig. S1). The microsatellite search was performed using the search parameters with the minimum repeat unit 12 for mono, 8 for di-; 5 for tri- and tetra-; 4 for penta- and hexa-nucleotides. Microsatellites were classified based on microsatellites locus length (ClassI > 20 and ClassII ≤ 20 nt)^43^ and nucleotide base composition of the microsatellites motif (AT rich, equal AT/GC content and GC rich). Microsatellites primers were designed with Primer3 with default parameters. Redundant primer sets were filtered using a Perl script (Table S12). SSR-containing flanking sequences were analysed with the ORF (open reading frame) finder Perl script using default parameters to predict the longest ORF within the SSR-containing flanking sequences. Then Augustus3^64^ was use to predict CDS (coding sequence), UTR (Untranslated regions) and introns regions in the flanking sequences. After that both ORF-finder output and Augustus output compile with the SSR position and assign the SSR locations. For, further verification the available genome annotations data of cv. 'Bedadeti' was retrieved from NCBI and compiled with SSR-location.

### Cross-taxa transferability, functional annotation and comparative mapping

In silico cross-taxa transferability of the enset to *Musa* was estimated using an e-PCR approach (permitting3 mismatches and 3 gaps). Predicted lengths of e-PCR amplicons were compared with the expected amplicon of each marker, if the length variation differs at least 6 bp, the markers were denoted as polymorphic. All the transferable markers were then mapped on the *Musa acuminata*^65^, *Musa balbisiana*^66^ and *Musa schizocarpa* genomes. Comparative mapping result was visualized by CIRCOS software^67^.

### Enset microsatellite marker database

To maximize the utility and availability of the enset microsatellite markers, we set up a searchable database using CSS, HTML and JavaScript under MySQL; a PHP based script was used to bridge the search interface and database, with results visualization, and download in XLS or CSV format.

### Tissue sampling, DNA extraction and PCR

Leaf samples of enset landraces were collected from across the distribution of enset in Ethiopia. Tissue samples were harvested from young cigar leaves and stored on silica gel. Genomic DNA was extracted using CTAB methods.

PCR amplification was performedfor microsatellite primer validation under the following conditions: 94 °C for 5 min, 35 cycles at 94 °C for 30 s, 56–60 °C (according to primer annealing temperature) for 30 s, and 72 °C for 45 s, followed by a final elongation at 72 °C for 5 min. PCR products were run on 1.5% agarose gels in 1 × Tris–Borate-EDTA (TBE) buffer and a 100-bp molecular ladder was used to estimate the amplicon size.

### Phylogeny and genetic diversity

A phylogenetic tree constructed based on SSR marker assay data, from the most highly polymorphic 15 EMM markers among 48 enset landraces and *Ensete* species. These fragment size variations were used for phylogenetic tree construction and subsequent genetic parameters analysis. Parameters including Polymorphism Information Content (PIC) of each marker; observed (H_o_) and expected (H_e_) heterozygosity; pair-wise comparisons of species genetic distance^68^ and F_ST_ (genetic differentiation) were calculated by PowerMarker version 3.25^69^. A Principal Coordinate Analysis (PCoA) was performed using the dissimilarity matrix data using GenAlEx software version6.5^70^. A dissimilarity matrix was estimated then transfered into Mega6 software^71^ and a Neighbor-joining (NJ) approach used to construct boot strap NJ-phylogenetic tree.

## Supplementary information

Supplementary file1.

Supplementary file2.

## Data Availability

Data generated in this study are included in the main table, figures, additional file and also deposited in the online portal with free accessibility (https://enset-project.org/EnMom@base.html or https://enset.aau.edu.et/index.html) and the database can be downloaded from https://figshare.com/s/20dd8c0d0a2994dbce8d.
