# Supplementary material for: The landscape of microsatellites in the enset (Ensete ventricosum) genome and web-based marker resource development
Source: Sci Rep. 2020 Sep 17;10:15312. doi: 10.1038/s41598-020-71984-x (PMC7498607; doi:10.1038/s41598-020-71984-x)
Supplement: Supplementary file 1 — Supplementary file1. [file 41598_2020_71984_MOESM1_ESM.docx]

**The landscape of microsatellites in the enset (*Ensete ventricosum*) genome and web-based marker resource development**

Manosh Kumar Biswas^1*^, Jaypal N. Darbar^1^, James S. Borrell^2^, Mita Bagchi^1^, Dhiman Biswas^3^,Gizachew Woldesenbet Nuraga^1,4^, Sebsebe Demissew^5^, Paul Wilkin^2^, Trude Schwarzacher^1^ and J. S. (Pat) Heslop-Harrison^1*^


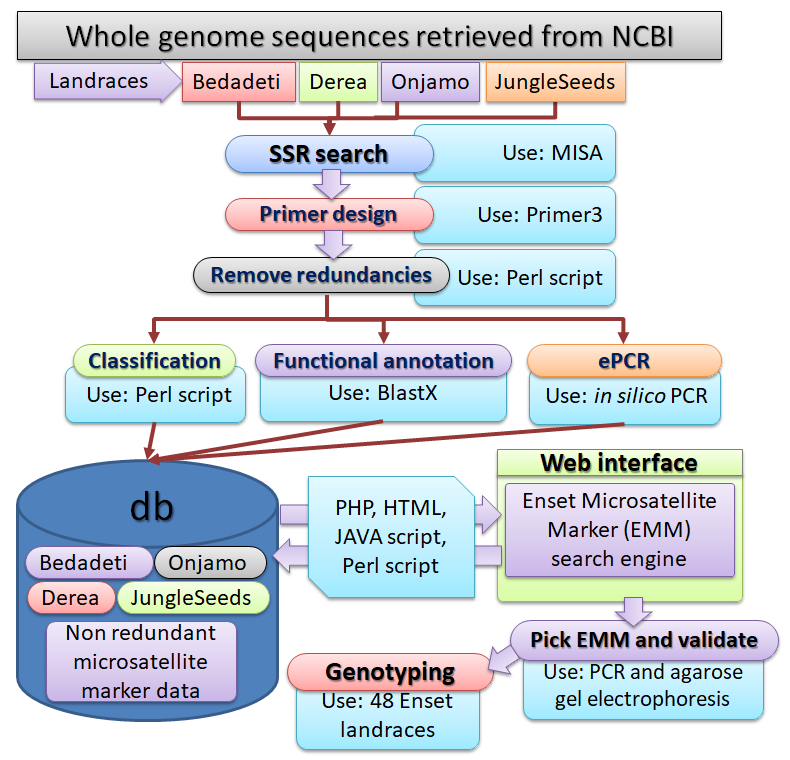


**Figure S1. Enset microsatellite analysis pipeline.** It includes genome sequences retrieved, microsatellite mining, microsatellite marker development, *in silico* validation, functional annotation, classification, marker database creation, microsatellite marker search interface development, wet-lab validation and utility for enset genotyping.

**
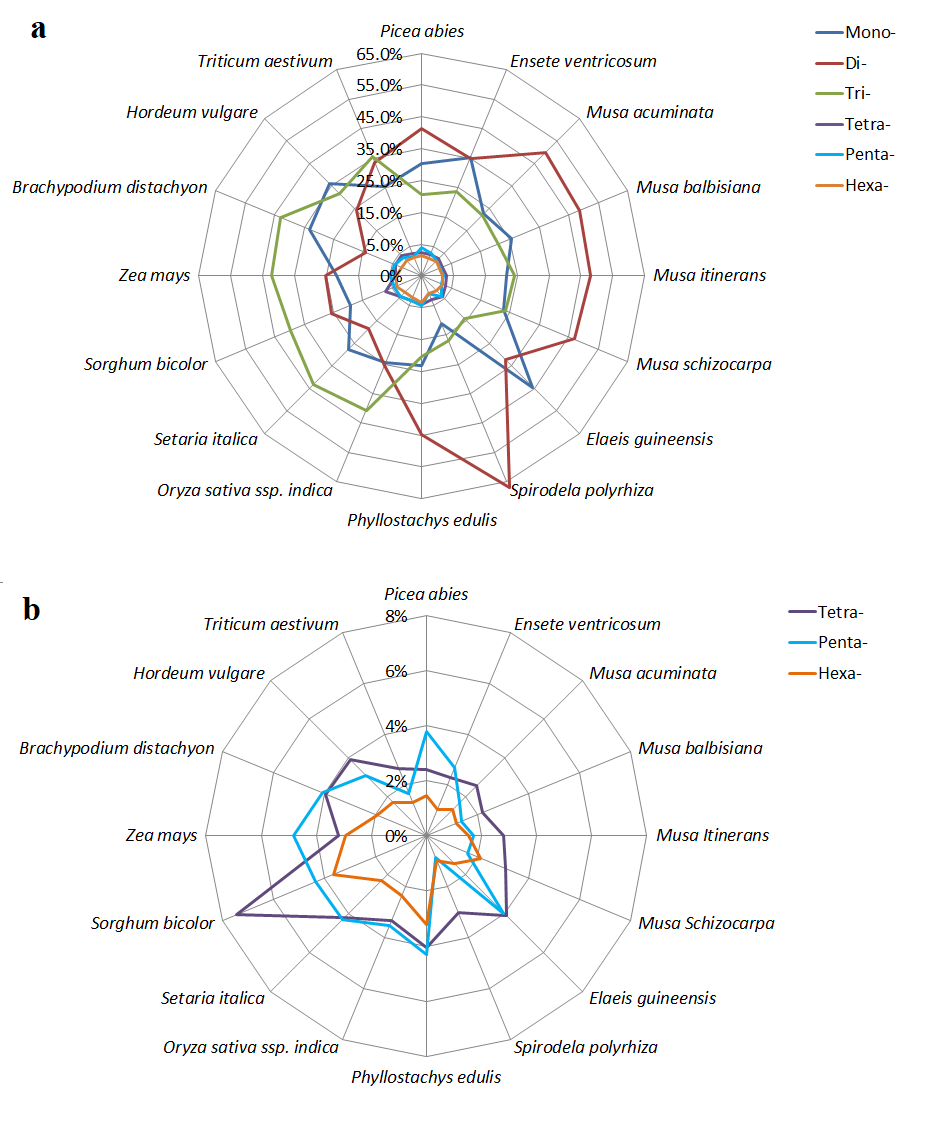
Figure S2. Presentation of the different repeat motif of microsatellite across the 16 plant species**. Displaying all motifs analysed (a) and enlarged (b) to show the differences in low frequency tetra- penta and hexa- nucleotides.


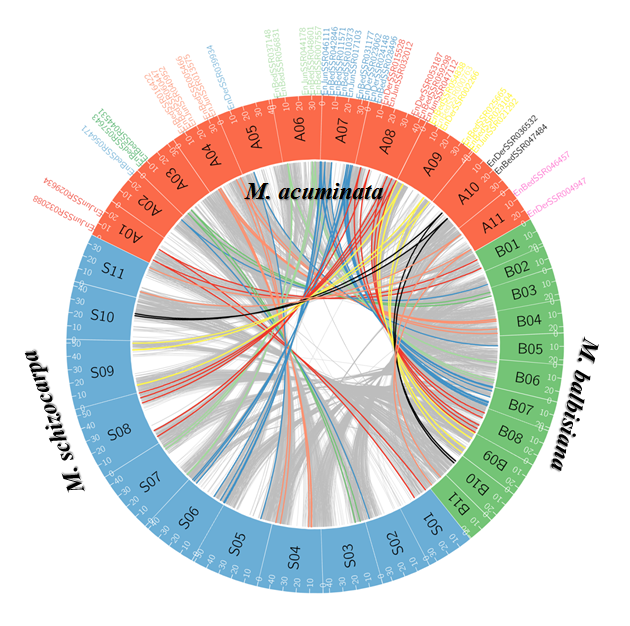


**Figure S3. Comparative mapping of Enset microsatellite markers (EMM) on chromosomes of Musa genomes.** A01 - A011: *M. acuminata* chromosome*,* B01 - B011: *M. balbisiana* chromosome and S01 - S11: *M. schizocarpa* chromosome. For data see Table S7. Each grey links represent one microsatellite marker position on the Musa genome. 40 EMM those were in vivo validated are presented here with different colour link and label with its marker id.


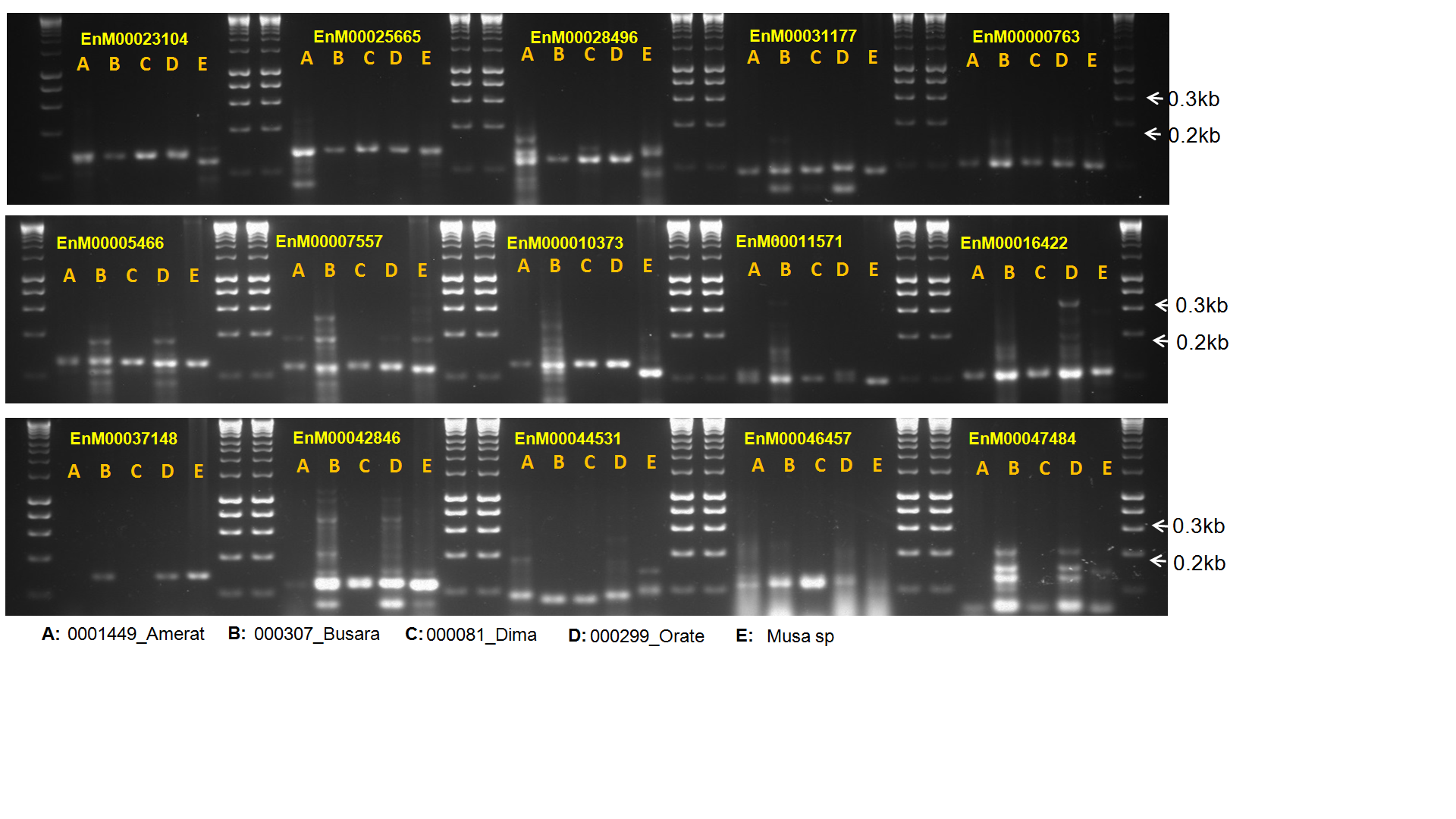


**Figure S4. PCR profile of the 15 *Ensete* microsatellite markers from their wet-lab validation.** Markers are: EnM00023104, EnM00025665, EnM00028496, EnM00031177, EnM00000763, EnM00005466, EnM00007557, EnM000010373,EnM00011571, EnM00016422, EnM00037148, EnM00042846, EnM00044531, EnM00046457, EnM00047484.


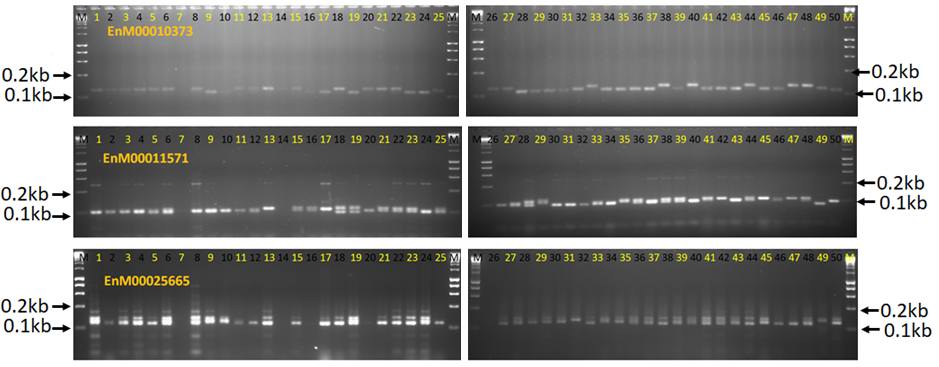


**Figure S5**. **PCR profile for 48 Enset landraces by three EMMs** (M: Hyper Ladder 1; lane 1-48 enset landraces, lane 49 and 50 as control use *Ensete* sp. and *Musa* sp.)
